# Conducting cross-cultural, multi-lingual or multi-country scale development and validation in health care research: A 10-step framework based on a scoping review

**DOI:** 10.7189/jogh.14.04151

**Published:** 2024-07-19

**Authors:** Yingxi Zhao, Richard Summers, David Gathara, Mike English

**Affiliations:** 1 Nuffield Department of Medicine Centre for Global Health Research, University of Oxford, Oxford, UK; 2 School of Social Policy, University of Birmingham, Birmingham, UK; 3 KEMRI-Wellcome Trust Research Programme, Nairobi, Kenya; 4 Centre for Maternal, Adolescent, Reproductive, and Child Health (MARCH), London School of Hygiene and Tropical Medicine, London, UK

## Abstract

**Background:**

Valid, reliable and cross-cultural equivalent scales and measurement instruments that enable comparisons across diverse populations in different countries are important for global health research and practice. We developed a 10-step framework through a scoping review of the common strategies and techniques used for scale development and validation in a cross-cultural, multi-lingual, or multi-country setting, especially in health care research.

**Methods:**

We searched MEDLINE, Embase, and PsycINFO for peer-reviewed studies that collected data from two or more countries or in two or more languages at any stages of scale development or validation and published between 2010–22. We categorised the techniques into three commonly used scale development and validation stages (item generation, scale development, and scale evaluation) as well as during the translation stage. We described the most commonly used techniques at each stage.

**Results:**

We identified 141 studies that were included in the analysis. We summarised 14 common techniques and strategies, including focus groups or interviews with diverse target populations, and involvement of measurement experts and linguists for item content validity expert panel at the item generation stage; back-and-forth translation, collaborative team approach for the translation stage; cognitive interviews and different recruitment strategies and incentives in different settings for scale development stage; and three approaches for measurement invariance (multigroup confirmatory factor analysis, differential item functioning and multiple indicator multiple causes) for scale evaluation stage.

**Conclusions:**

We provided a 10-step framework for cross-cultural, multi-lingual or multi-country scale development and validation based on these techniques and strategies. More research and synthesis are needed to make scale development more culturally competent and enable scale application to better meet local health and development needs.

Scales measure latent constructs and specifically ‘behaviours, attitudes, and hypothetical scenarios we expect to exist as a result of our theoretical understanding of the world, but cannot assess directly’ [1]. Developing a new scale could help us measure a more specific behaviour and experience. However, scale development and validation processes can be long and complex, usually involving three stages: item development, where an initial item pool is produced via deductive, inductive, or combined approaches; scale development, where individual items are constructed into harmonious constructs; and scale evaluation, where the validity and reliability of the scale are tested [2–4].

Global health often needs valid, reliable, and universally applicable tools and measures that enable comparisons across culturally diverse populations in different countries [5–7]. However, measurement instruments developed in one context are often translated into other languages or taken into other contexts without proper equivalence assessment [8]. When evaluated, the psychometric properties in new settings often suggest poor performances [9]. Poor translation quality can contribute to this. Still, cultural differences exist in the conceptual and operational definitions of many behaviours and experiences being measured, which can lead to measurement inequivalence [10,11]. If the aim is to produce comparable and generalisable evidence across contexts, we need to consider how to develop a scale that performs well in different cultures, countries and languages right from the start.

Here, we extend Boateng et al. [1] framework for scale development and validation to encompass such cross-context concerns through a scoping review. Our research question was, ‘What are common strategies and techniques that have been used for scale development and validation in a cross-cultural, multi-lingual or multi-country setting, especially in health care research?’ We then extended the Boateng et al. framework to 10 steps to incorporate these considerations. We hope this 10-step framework may help researchers who aim to develop a new scale for use in different settings or those who aim to adapt an existing scale to a new language or country.

## METHODS

We followed the five steps of the Arksey and O’Malley method [12] for scoping reviews to identify studies that conducted scale development and/or validation in a cross-cultural, multi-lingual, or multi-country setting.

### Search strategy and screening

We conducted a systematic search using MEDLINE, Embase, and PsycINFO to obtain relevant articles in health care research, using search terms such as multi-country, cross-cultural, and multigroup (Table S1 in the **Online Supplementary Document**). We included empirical studies published between 2010–22 in English due to time and resource constraints. We included studies if they reported data collection from two or more countries or in two or more languages at any stages of scale development or validation, for example, if a scale was first developed in English and then validated in several other languages or countries. We excluded studies that validated a previously developed scale in a new sample but only in one single language or country; we also excluded studies that focused on measurement invariance of different population groups with different culture or socio-demographic characteristics within one country, despite that some of our findings would still be of relevance for those studies. After deduplication, one reviewer conducted two stages of title/abstract and full-text screening in Abstrackr (Brown University Center for Evidence Synthesis in Health, Providence, Rhode Island, USA) [13] and Microsoft Excel, version 16 (Microsoft Corporation, Redmond, Washington, USA), respectively. Uncertainty was resolved through discussions with other co-authors.

### Data charting, collation and reporting

We further charted data from the articles that were included and entered them into a Microsoft Excel spreadsheet. We charted the following data: title, authors, survey sample size, survey country number, survey language number, and technique for cross-cultural, multi-lingual, or multi-country scale development and validation. We categorised the technique into three stages, as listed in Boateng et al. [1], which included item development, scale development, and scale evaluation. We also charted the strategy used for translation when more than one language was used. When available, we extracted these data by looking into the methods, results, tables/figures, and questionnaire appendices. For reporting of our findings, we described the most commonly used techniques at each stage, i.e. used more than once in our included studies, and where relevant, traced back to the cited methodology papers when the authors referenced specific techniques they used. We organised these techniques and strategies and extended the framework by Boateng et al. [1] into a 10-step framework for cross-cultural, multi-lingual, or multi-country scale development and validation.

## RESULTS

Of the 1985 citations identified after deduplication, 141 met inclusion criteria after the full-text review. The included studies had an average 5220 sample size in their survey administration stage, 134 included more than one country in their sample, and 102 used more than one language (Figure S2 and Table S3 in the **Online Supplementary Document**).

**Figure 1:**
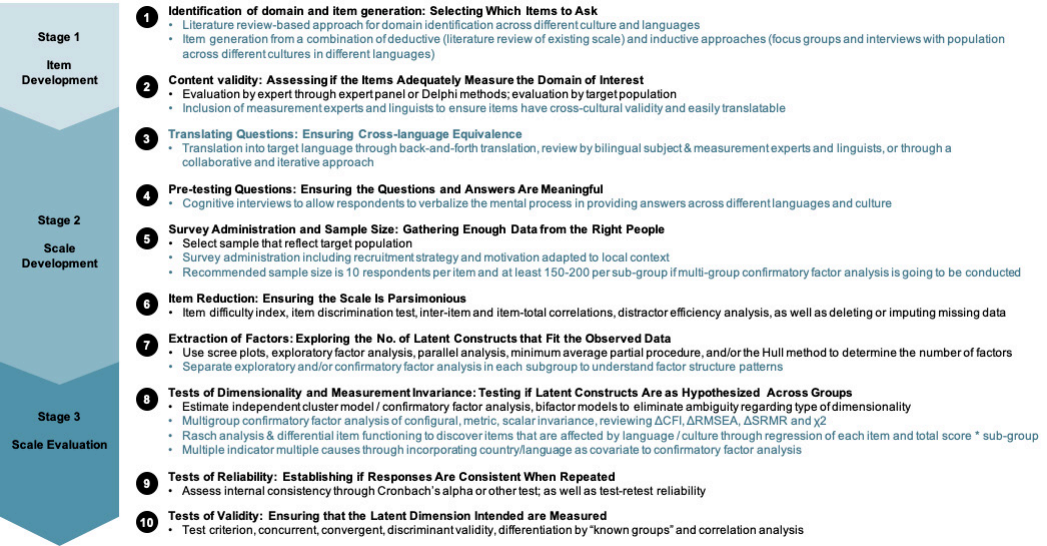
A 10-step cross-cultural, multi-lingual or multi-country scale development and validation framework. Adapted from the nine-step scale development and validation framework by Boateng et al. with added recommendations for cross-cultural, multi-lingual or multi-country scale development and validation in blue.

**Table 1** summarises the technique or strategy used by more than one study at each scale development and validation stage. **Figure 1** further extends the scale development and validation framework by Boateng et al. [1] to incorporate these techniques and strategies for cross-cultural, multilingual, or multi-country settings. In the following section, we explain each technique and provide examples of their use. Readers could also refer to the references listed in **Table 1** for more detail.

**Table 1:** Commonly used techniques and strategies for cross-cultural, multi-lingual or multi-country scale development and validation

Technique and strategy	Description		Publications that reported its use (n)		Examples
Item development stage					
*Literature based reviews to capture existing tools or constructs*	Literature search in databases to capture relevant validated instruments across different countries and settings.		8		Bloemeke et al. [14], Perkmen et al. [15]
*Individual concept elicitation or in-depth interviews with target population*	Exploratory interviews in different countries and settings.		6		Kerrigan et al. [16], Chen et al. [17]
*Focus group discussions with target population*	Focus group discussions in different countries and settings to allow respondents to explore and clarify individual and shared perspectives.		9		Aizpitarte et al. [18], Luquiens et al. [19]
*Expert panel or consensus group*	Inputs from subject experts, measurement experts, and linguists to provide or review items have cross-cultural validity and will be easily translatable.		8		Nackers et al. [20], Abraham et al. [21]
Translation stage					
*Back-and-forth translation*	First translate from source language to target language, then back translate to source language by a second translator, and lastly compare and resolve inconsistency.		63		Mezquita et al. [22], Roberts et al. [23]
*Expert review*	Translated items reviewed by bilingual subject experts, measurement experts and linguists.		11		Wilson et al. [24], Vogel et al. [25], Vaingankar et al. [26]
*Collaborative and iterative translation*	A collaborative approach through parallel or double translation, pretesting, and revise. No back-translation.		3		Hakim and Liu [27], Sproesser et al. [28]
Scale development stage					
*Cognitive debriefing or interview*	Pilot participants asked their understanding of each instruction, item and response options to evaluate the interpretation and acceptability; sometimes followed by questions on general perception of the draft scale.		8		Luquiens et al. [19], McCoy et al. [29]
*Different ways of recruitment*	Survey administration including recruitment strategy and motivation should be adapted to local context and logistics feasibility.		2		Korf et al. [30], O’Brien et al. [31]
*Separate reliability test in each sample*	Cronbach’s α-based reliability analysis and item-to-scale correlational analyses in each sample.		3		Littrell et al. [32], Whiting-Collins et al. [33]
*Separate factor analysis in each sample*	Separate exploratory and/or confirmatory factor analysis in each sample to understand the factor structure patterns between different samples. For confirmatory factor analysis, commonly reported model fit indices are CFI>0.90 or CFI>0.95, RMSEA<0.08, SRMR<0.08, and Tucker–Lewis index >0.90.		30		Encantado et al. [34], Stevelink et al. [35]
Scale evaluation stage					
*MGCFA*	Technique under classical test theory. Three most commonly used measurement invariance are configural invariance (same number of factors and pattern of loading), metric invariance (factor loading across groups), scalar invariance (same item intercepts); and commonly reported invariance indices are ΔCFI (commonly below 0.01), ΔRMSEA (commonly below 0.015), ΔSRMR (commonly for metric level below 0.03) whereas others also used the χ^2^ χ^2^ test.		84		Datu et al. [36], Lopez-Fernandez et al. [37]
*Rasch analysis and DIF*	Technique under item response theory. DIF is useful to discover with item function differently across sub-groups, through having each item as the dependent variable and the total score and sub-group as well as their interaction as covariates, and significant changes in coefficient of determination indicate response to the examined item is affected by language or country.		19		Erhart et al. [38], Lau et al. [39], Geyh et al. [40]
*MIMIC*	Confirmatory factor analysis with sub-group (country or language) as covariate, and significant direct effect on model indices suggest inequivalence.		3		Boudjemadi et al. [41], Pendergast et al. [42]

CFI – comparative fit Index, DIF – differential item functioning, MGCFA – multi-group confirmatory factor analysis, MIMIC – multiple indicator multiple causes, RMSEA – root-mean-square error of approximation, SRMR – standardised root mean square residual, ΔCFI – change in comparative fix index, ΔRMSEA – change in root-mean-square error of approximation, ΔSRMR – change in standardised root mean square residual

### Item development stage (steps one and two)

For step one, which is generating the domain and individual items, techniques that considered cross-cultural, multi-lingual, or multi-country equivalence included literature reviews to capture constructs of interest or existing tools that measure similar phenomena or focus groups and individual interviews to generate items inductively with the target population, which were usually conducted in multi-country settings to generate items that could be broadly generalisable. For example, Chen et al. [17], when developing a Family Role Performance Scale, conducted interviews with 15 Israeli and 11 United States respondents with a diverse array of family structures. They started with general questions about family roles and then moved on to specific questions, using a funnel approach. The interview transcripts were translated and coded for concepts of interest, further informing their categorisation of 17 items.

At the content validity step, which focused on whether the items adequately measure the domain (step two), some reported using expert panels or consensus groups to review their items, and subject experts were deemed important in ensuring all relevant topics were covered in the items. In Abraham et al. [21] development of an oestrogen plus progestin therapies-related breast symptoms questionnaire, after items were generated from concept elicitation interviews with the target population, a group of measurement and clinical experts and linguists reviewed their items. The inclusion of linguists specifically ensured that items had cross-cultural validity, could be easily translated into the four countries of study, and were conceptually equivalent in other potential languages.

Other approaches at this stage reported in literature included: 1) Delphi technique by Michaud et al. [43] in their item generation stage, where participants were asked to discuss and rate different concepts of interest, 2) Benschop et al. [44] when designing a new tool for understanding new psychoactive substance use in six European countries, conducted extensive literature-based review, country reports and expert consultation to understand the policy and context of substance use, which informed their item generation, 3) O’Brien et al. [31] also reported that their research team had members with diverse culture, different ages and experiences which helped to reduce biases.

### Translation (step three)

Out of 118 studies that reported using more than one language for their measurement scale, 80 reported their translation strategy. While very few studies used one-way translation (from the source language to the target language), most used back-and-forth translation techniques, and some also specifically referenced the Brislin procedure for back-and-forth translation, which included three steps of translating into the target language, back-translating into source language by another translator and then resolving inconsistency through discussion [45]. In other studies, researchers reported the use of expert reviews after translation. For example, after translating the draft scale to Mandarin, Wilson et al. [24] invited psychologists with measurement and construct expertise to ensure lexical and construct equivalences were maintained. Some studies also used collaborative and iterative translation, or the committee method, which is considered an alternative to back-translation to ensure better conceptual equivalence [27,28,46]. Several other studies translated their instruments following specific organisational guidelines [47,48].

### Scale development stage (steps four to seven)

In the scale development stage, items are selected and translated, and data are collected. However, there are other factors to consider in the data collection process. For pre-testing (step four), several studies conducted cognitive interviews or cognitive debriefings to ensure their scale’s face and content validity. For example, Luquiens et al. [19] asked patients in their study countries to complete their draft scale. Then, they asked them questions to assess their understanding of each instruction, item and response option. This led to the removal of 14 items that were considered redundant, ambiguous, or difficult to understand, as well as eight revised items.

For scale administration (step five), two studies reported different ways of recruitment. In O’Brien et al. [31] study, where they recruited South Korean and White mothers, the former received small-amount gift cards for a cup of coffee, whereas the latter had the opportunity to enter a lottery to win a larger prize due to cultural differences regarding incentives. Benschop et al. also used different strategies to recruit new psychoactive substance users via nightclubs, drug services and Facebook groups based on differences in country settings [30,44]. These considerations could also be relevant to earlier stages of recruitment for focus groups or cognitive interviews. Additionally, there are other considerations for sample size, both linked with overall scale development and multi-group confirmatory factor analysis (MGCFA), discussed below at the scale evaluation stage (step eight).

For the psychometric analysis of the scale development stage (steps six and seven), several studies highlighted the first use of internal reliability and item correlational analysis in each sample separately. More commonly, studies reported conducting separate exploratory and/or confirmatory factor analyses in each sample to understand the factor structure patterns between different samples. For example, Bothe et al. [49] ran a confirmatory factor analysis on their five theory-based factors and 19 representing items in four separate samples, all of which had acceptable model fits. This step is usually required before running MGCFA in the evaluation stage. In Stevelink et al. [35], an exploratory factor analysis of participants from six countries was performed per database, suggesting two different factor structures (one-factor vs two-factor model). They further conducted MGCFA using both structures, and the two-factor model suggested a better overall fit. For the studies that conducted confirmatory factor analysis and provided model fit indices, four indices are commonly reported – comparative fit index (CFI>0.90 or CFI>0.95), root-mean-square error of approximation (RMSEA<0.08), standardised root mean square residual (SRMR<0.08) and Tucker–Lewis index (TLI>0.90) (Table S4 in the **Online Supplementary Document**).

### Scale evaluation stage (steps eight to 10)

All the studies that reported different techniques used at the evaluation stage focused on cross-country or cross-language measurement invariance, i.e. whether the psychometric properties are generalisable across different sub-groups (step eight). The most commonly used technique is MGCFA. MGCFA usually examines three types of measurement invariance, i.e. configural invariance (same number of factors and pattern of loading), metric invariance (factor loading across groups), and scalar invariance (same item intercepts). However, others also examined strict invariance (invariance of the item residuals) [36,37]. For studies that reported invariance indices, three indices are commonly used: change (Δ) in CFI<0.01, ΔRMSEA<0.015, and ΔSRMR<0.03 (commonly for the metric level and for scalar level there is no consensus). Chen [50] and Cheung and Rensvold [51] are the most commonly cited references for these cut-off values. Several other studies also used χ^2^ test statistic or alignment methods. For the 83 studies that provided sample size by group for MGCFA, 70 had more than 150 participants per group, and 63 had more than 200 per group (Table S4 in the **Online Supplementary Document**). For example, in Lopez-Fernandez et al. [37], cross-cultural validation of the Compulsive Internet Use Scale across eight languages, configural invariance was supported. However, ΔCFI and ΔRMSEA between the metric and scalar model exceeded cut-off thresholds. Therefore the factor structure and loading were invariant between eight languages. Still, the latent factor means and residuals could be different.

While MGCFA fits under the classical test theory, others used Rasch analysis and differential item functioning (DIF), which are item-response theory techniques. The Rasch model is most useful in evaluating individual items’ functioning as it estimates both scale and item-level fit indices. Within Rasch analysis, DIF is commonly used to examine cross-cultural validity across subgroups. Usually, each item is examined as a dependent variable with the total score and sub-group (language or country) and their interaction as covariates. Significant changes in the coefficient of determination suggest significant DIF, i.e. response to the examined item is affected by language or country which could help select items that should be re-considered for cross-cultural invariance. For example, Geyh et al. [40] conducted DIF analyses using data from four countries. They highlighted two items with DIF in the Satisfaction with Life Scale and, through Tukey-Cramer post-hoc tests, highlighted that data from Israel showed the most frequent differences from the other countries.

Lastly, several studies also reported using multiple indicator multiple causes (MIMIC), a variant of confirmatory factor analysis that further incorporated covariates. One paper also claimed that MIMIC is more appropriate for analyses across many groups [42]. Boudjemadi et al. [41], in their testing of multigroup invariance across countries, added a country covariate to their MGCFA model. They observed a significant direct effect of the country covariate on the model fit indices and concluded that factor means behave differently across countries. Besides these three commonly used techniques, other papers also reported using approaches such as the sequential constraint imposition [52] and Satorra-Bentler χ^2^ difference test [27].

We did not identify specific techniques used for steps nine and 10, which are the reliability and validity tests.

## DISCUSSION

Conducting cross-cultural, multi-lingual or multi-country scale development and validation represents unique logistics and analytical challenges. In this scoping review, through reviewing and analysing 141 published cross-setting, scale development, and validation articles, we summarised the common techniques and strategies used to ensure that the scale has cross-lingual or cross-country equivalence. While a protocol has been published on adapting measurement scales to a new context [11], our review further extends this to developing new scales across contexts often needed for global health research and practice.

In **Figure 1**, we have provided a 10-step framework of cross-cultural, multi-lingual or multi-country scale development and validation extending the original nine-step framework by Boateng et al. [1]. This framework is based on published health care research papers but is also relevant to other disciplines such as psychology, management and education. Some steps apply to broader global health research and practices, such as conducting multi-country surveys. While all the techniques listed could further improve scale development and validation rigour, researchers might be constrained by resources such as time and funding and have to decide which steps and techniques to prioritise. In the following sections, we highlight some of the key considerations for researchers.

Prior to scale development for cross-cultural settings, there is a need to ensure that the measure or phenomenon truly exists as a culturally independent construct and that the construct itself can be robustly measured [53]. This could be achieved by techniques listed in steps one and two (**Figure 1**), e.g. literature-based review and qualitative work with the target population across settings. Having a diverse research team [31] and understanding the policy context through examining policy documents and conducting expert consultations are also useful [44]. Occasionally, some measures, constructs or items are not universally relevant but of great importance to certain settings, in those cases slightly different versions of a scale could be developed for different settings.

Commonly draft scales need to be translated into additional target languages (as shown in step three). While most papers reported back-and-forth translation, how developers handled the inconsistency between the source and back-translated versions is unclear. We agree with Douglas et al. that reliance on back-translation could be problematic as bilingual translators could make sense of a poorly written target translation. There are subtle nuances in the use of languages and idioms [54]; therefore, using a team-based approach and the involvement of measurement experts and linguists could help discover these issues [54,55]. We also highlight the importance of conducting cognitive interviews in pilot testing (step four). Cognitive interviews could significantly improve the validity of surveys in global health settings. This is because during the actual survey scale, instructions and items could be understood in unexpected ways, and item responses could be considered inappropriate, compromising the study quality [56]. Unfortunately, only eight studies in our 141 included articles mentioned cognitive interviews in different settings, which might be related to the lack of emphasis on this methodology in global health surveys that rely more on quantitative approaches. We strongly recommend using cognitive interviews for cross-setting scale development, and more details on conducting cognitive interviews can be found in Scott et al. [56]

Measurement invariance is often the centrepiece for cross-setting scale development and validation, as most papers reported using one statistical approach for invariance analysis (step eight). Despite many critiques, MGCFA is still the most commonly used and ‘the most powerful and versatile’ approach [57,58]; therefore, we recommend using MGCFA if the aim is to produce a universally applicable scale for cross-cultural settings. We acknowledge that there are different invariance indices used in our review, including ΔCFI, ΔRMSEA, and ΔSRMR, as well as slight inconsistency on cut-offs (especially whether, for example, ΔCFI should be <0.01 or ≤0.01) (Table S4 in the **Online Supplementary Document**), and some others also used the more restrictive change in χ^2^ test. We recommend reporting three indices. As a relaxed fit, ΔCFI≤0.01, ΔRMSEA≤0.015, and ΔSRMR≤0.03 at the metric level. Aside from MGCFA, if the aim is to identify specific non-equivalent items, researchers should use DIF. If there are many sub-groups in measurement invariance analysis, researchers should consider MIMIC [42].

Regarding sample sizes for step five on survey administration, various rules have been suggested for determining the sample size for the questionnaire survey and scale development. The general rule of thumb for scale development is 10 participants per item [1,59]. For multi-lingual or multi-country scale development where MGCFA is going to be conducted, researchers need to be mindful of whether the sample size in the sub-group is sufficient to obtain an accurate factor solution. Guadagnoli suggested that the actual number is dependent on the number of items per scale construct and component saturation (the magnitude of component loading), and if there are 10 or more items representing each construct, a sample size of 150 should be sufficient [60], and Hair et al. recommended a sample of 200 [61]. Most of our included studies had more than 150 or 200 samples in their smallest subgroup. Therefore, we recommend that researchers use both criteria when determining sample size if MGCFA is to be performed (10 participants per item for the overall sample, and 150–200 samples per sub-group). There are no established guidelines on the sample size required for Rasch and DIF analyses, and 100–200 per subgroup is commonly recommended [62–64].

Developing and administering the scale in multiple countries and languages could also bring logistic and sometimes ethical challenges. Challenges to find and acknowledge the roles of translators, interviewers and survey staff [65], standardisation of recruitment strategies [66], as well as the ethical challenges such as the power asymmetries between the scale development team and frontline staff and whether the scale being developed is relevant and prioritised by local population [67,68] should all be taken into consideration. Our reviewed papers also provided examples of how to recruit in diverse settings and what motivation incentives should be in place [30,31,44]. Researchers could also refer to the broad literature on cross-cultural research when conducting multi-lingual and multi-country scale development [65-67,69–71].

Several limitations should be considered for this review. First, screening and data charting are conducted by only one reviewer. While there could be bias in the selection and extraction process, the reviewer has rich experiences in different review types; thus, the risk of bias is relatively lower. Second, as this is a scoping review, we did not aim to systematically assess the quality of included studies but only reported on what techniques and strategies have been used. Lastly, it should be noted that our inclusion and exclusion criteria might have led to biases. For example, we included studies that included two or more countries or two or more languages in their scale development, and we excluded studies that focused on measurement invariance of different population groups with different cultural or socio-demographic characteristics within one country; inclusion of these studies might have identified additional techniques. An example of examining measurement invariance across culture and socio-demographics could be found in Dong and Dumas [72]. We also only focused on papers published in health care journals in English due to time and resource limitations. While the authors include researchers from different disciplines (health care, medicine, nursing, psychology) and three authors also come from global health backgrounds, which improved the review’s relevance and comprehensiveness, we acknowledge that further extension to related fields such as education and psychology journals, non-English language papers, and the inclusion of papers that investigated cultural differences within one country and language could help strengthen our recommendations.

## CONCLUSIONS

Scale development and validation in cross-cultural, multi-lingual or multi-country settings is important for global health research and practice, but the process could be challenging. Reviewing current techniques and strategies in published scale development papers, we summarised key recommendations at item generation, translation, scale development, and scale evaluation stages. We produced a newer, expanded 10-step scale development and validation framework (**Figure 1**). As scale development is complicated and requires multiple iterations, and universal and cross-cultural equivalence is always more a goal than a reality, this review should be considered a ‘jumping off point’ for anyone interested. More research and synthesis are needed to make scale development more culturally competent and enable scale application to better meet local health and development needs.

## Additional material


Online supplementary document
