# Supplementary material for: Conducting cross-cultural, multi-lingual or multi-country scale development and validation in health care research: A 10-step framework based on a scoping review
Source: J Glob Health. 2024 Jul 19;14:04151. doi: 10.7189/jogh.14.04151 (PMC11257704; doi:10.7189/jogh.14.04151)
Supplement: Online supplementary document [file jogh-14-04151-s001.pdf]

Appendix S1. Search strategy

Database(s): Embase 1974 to present, Medline (Ovid MEDLINE® Epub Ahead of Print, In-Process & Other Non-Indexed Citations, Ovid MEDLINE® Daily and Ovid MEDLINE®) 1946 to present, PsycINFO 1806 to present
Search Strategy:

#	Searches	Results
1	(scale* or index* or tool* or instrument*).ti.	908523
2	(develop* or valid*).ti.	2073973
3	("multilanguage" or "multi-language" or "multicountry" or "multi-country" or "multigroup" or "multi-group" or "cross language" or "cross-language" or "cross country" or "cross-country" or "cross cultur*" or "cross-cultur*" or "across cultur*" or "across setting*").tw.	115450
4	1 and 2 and 3	4667
5	limit 4 to (english language and yr="2010 - 2022")	3683
6	remove duplicates from 5	1985

Appendix S2. PRISMA Diagram

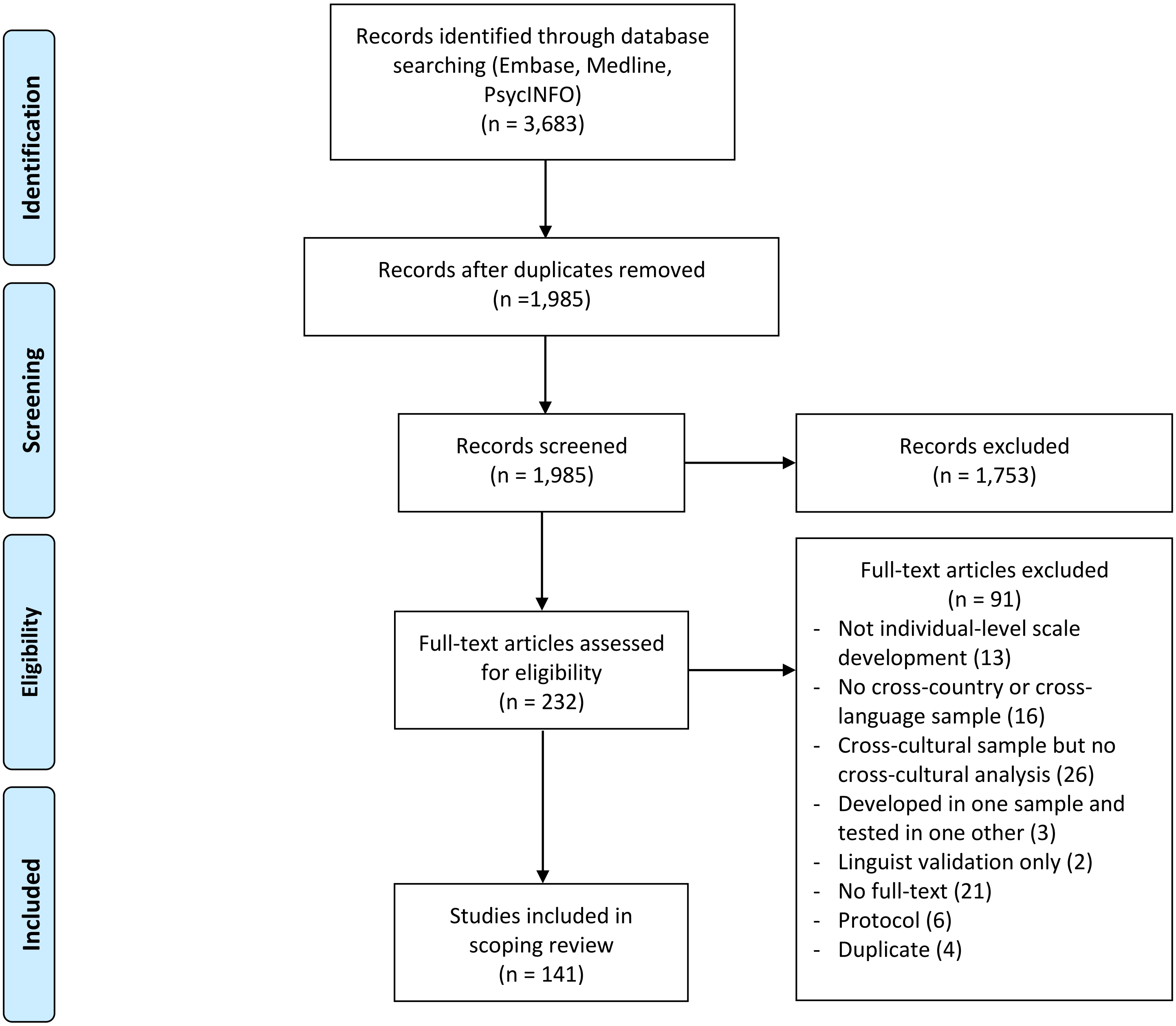

Appendix S3. Detail of included studies

First author	title	journal	Item development	Technique used	Scale development	Technique used	Translation	Scale validation	Technique used	Country number	Language number	Survey sample size
Benschop A.	Why do people use new psychoactive substances? Development of a new measurement tool in six European countries	J. Psychopharmacol.	X	Research on policy and context in each country; Expert consultation	X	Different way of recruitment in each country	Back and forth translation	X	MGCFA	6	6	3023
Heckert J.	Development and validation of a health and nutrition module for the project-level Women's Empowerment in Agriculture Index (pro-WEAI+HN)	Matern Child Nutr	X		X			X	MGCFA	3	N/A	12114
Taris, Toon W	Going your own way: A cross-cultural validation of the Motivational Demands at Work Scale (mind@work).	Frontiers in Psychology					Back and forth translation	X	MGCFA	2	2	989
O'Brien, Karen M	The Good Mothering Expectations Scale: An international instrument development study.	The Counseling Psychologist	X	Review to capture constructs; multi-cultural research team member	X	Separate EFA and CFA; Difference in incentives	Back and forth translation			2	2	1238
Wilson C.A..	A cross-cultural validation of the Resiliency Scale for Young Adults in Canada and China	Psych J					Expert review translation	X	MGCFA	2	2	1268
Datu J.A.D.	Psychometric validity and measurement invariance of the caring for Bliss Scale in the Philippines and the United States	J Am Coll Health						X	MGCFA	2	1	1189
Lawson, Jessica L	Cross cultural validation of the Yale Food Addiction Scale 2.0 and evaluation of food addiction in Hispanic bariatric surgery-seeking patients.	Dissertation Abstracts International: Section B: The Sciences and Engineering					Back and forth translation	X	MGCFA	1	1	444
Roberts M.E.	Testing the Situationism Scale in Europe: Scale validation, self-regulation and regional differences	Int J Psychol					Back and forth translation	X	MGCFA	23	6	1106
Walker, Timothy J	Examining the validity, reliability, and measurement invariance of the Social Support for Exercise Scale among Spanish- and English-language Hispanics.	Hispanic Journal of Behavioral Sciences					Back and forth translation	X	MGCFA	1	2	1447
Aizpitarte A	Development of a Dating Violence Assessment Tool for Late Adolescence Across Three Countries: The Violence in Adolescents' Dating Relationships Inventory (VADRI)	J Interpers Violence	X	Interviews and FGD to generate items; response considering country difference	X	Cognitive interview		X	differential item functioning (DIF) using multiple regression	3	1	466
Wanstrom, Linda	Perceived collective efficacy to stop aggression at school: A validation of an Italian and a Swedish version of a scale for adolescents.	European Journal of Developmental Psychology					Back and forth translation	X	MGCFA	2	2	678
Kahn, Dennis T	Measuring parental anchoring: The development and validation of the Parental Anchoring Scale.	TPM-Testing, Psychometrics, Methodology in Applied Psychology					Back and forth translation	X	MGCFA	2	2	472
Perkmen, Serkan	Cross-cultural search for Big Five: Development of a scale to compare personality traits of pre-service elementary school teachers in Turkey and Spain.	Asia Pacific Education Review	X	Review to capture similar scales in each language and culture	X	Separate PCA and CFA in each language				2	2	555
Schnettler, Berta	Cross-cultural measurement invariance in the Satisfaction with Food-Related Life Scale in older adults from two developing countries.	Health and Quality of Life Outcomes						X	MGCFA	2	1	1573
Monaco E.	Cross-cultural validation of the Worries about COVID-19 and its consequences Scale (W-COV) in adolescents and young people	Arch Psychiatr Nurs						X	MGCFA	5	1	5559

Hakim, Moh Abdul	Development, construct validity, and measurement invariance of the Parasocial Relationship with Political Figures (PSR-P) Scale.	International Perspectives in Psychology: Research, Practice, Consultation					Committee approach	X	Satorra-Bentler χ2 (SB -χ2) difference test	3	2	1399
Erhart M.	A comparison of Rasch item-fit and Cronbach's alpha item reduction analysis for the development of a Quality of Life scale for children and adolescents	Child Care Health Dev						X	DIF	7	1	3019
Brunet J.	Measurement invariance of English and French Health Education Impact Questionnaire (heiQ) empowerment scales validated for cancer	Qual. Life Res.					Hawkins and Osborne	X	MGCFA	2	2	1224
Ruvio, Ayalla A	Consumer arrogance: Scale development and validation.	Journal of Business Research						X	MGCFA and nomological medel (SEM)	2	1	347
Abraham L.	Qualitative cross-cultural exploration of breast symptoms and impacts associated with hormonal treatments for menopausal symptoms to inform the development of new patient-reported measurement tools	Maturitas	X	Interview and open-end questioning/concept elicitation interviews; input from measurement experts, clinical experts and linguists						4	4	59
Gagne, Marylene	The Multidimensional Work Motivation Scale: Validation evidence in seven languages and nine countries.	European Journal of Work and Organizational Psychology	X		X		Back and forth translation	X	MGCFA	9	7	3435
Littrell, Romie Frederick	Explicit preferred leader behaviours across cultures: Instrument development and validation.	Journal of Management Development			X	Separate SEM in each country	Brislin			16+	10	N/A
Kerrigan D.	Development of the Experiences of Sex Work Stigma Scale Using Item Response Theory: Implications for Research on the Social Determinants of HIV	AIDS Behav	X	In-depth interview	X	Cognitive debriefing interviews		X	DIF	2	2	416
McCoy, Dana Charles	Measuring early childhood development at a global scale: Evidence from the Caregiver-Reported Early Development Instruments.	Early Childhood Research Quarterly	X	Review of tools	X	Cognitive interview	Back and forth translation			17	N/A	8022
Mccusker P.J.	International cross-cultural validation study of the Canadian Haemophilia Outcomes: Kids' Life Assessment Tool	Haemophilia			X	Cognitive debriefing interview	Back and forth translation, expert review			5	5	265
Admi H.	Stress measurement among charge nurses: developing a cross-cultural tool	J Adv Nurs			X	Separate CFA				3	3	2616
Martin-Fernandez, Manuel	Refinement of the Analysis-Holism Scale: A cross-cultural adaptation and validation of two shortened measures of analytic versus holistic thinking in Spain and the United States.	Personality and Individual Differences			X	Cross-cultural expert review of items	Back and forth translation, expert review	X	MGCFA	2	2	2254
Oort Q.	Development of an EORTC questionnaire measuring instrumental activities of daily living (IADL) in patients with brain tumours: Phase I-III	Neuro-Oncology	X	literature review	X	Cognitive debrefing	EORTC Quality of Life Group Translation Procedure			5	5	129
Metin, U. Baran	Validation of the Procrastination at Work Scale: A seven-language study.	European Journal of Psychological Assessment					Back and forth translation	X	MGCFA	7	7	1028
Caqueo-Urizar, Alejandra	Validation of the GEOPTE scale of social cognition for psychosis in patients diagnosed with schizophrenia in Latin America.	Behavioral Psychology						X	MGCFA	3	1	253
Mourao, Luciana	Professional development short scale: Measurement invariance, stability, and validity in Brazil and Angola.	Front Psychol					Borsa	X	MGCFA	2	1	2547

Leger R.R.	Stigma in adults with sickle cell disease and family members: Scale development and pilot study in the USA and Nigeria	Int. J. Afr. Nurs. Sci.	X	Individual check cultural competency and content validity						2	1	42
Cid, Luis	Cross-cultural validation of the Basic Psychological Needs in Physical Education Scale between Portugal and Brazil samples.	The Spanish Journal of Psychology						X	MGCFA	2	1	1066
Arana F.G.	Cross-Cultural Validity of the Ruminative Responses Scale in Argentina and the United States	Assessment						X	Nested model comparison	2	2	679
Rahafar A.	Cross-cultural validity of Morningness-Eveningness Stability Scale improved (MESSi) in Iran, Spain and Germany	Chronobiol. Int.					Back and forth translation	X	MGCFA	3	3	716
Bothe B.	The development of the Compulsive Sexual Behavior Disorder Scale (CSBD-19): An ICD-11 based screening measure across three languages	J. Behav. Addict.			X	Separate CFA	Back and forth translation	X	MGCFA	3	3	9375
Velten, Julia	Positive Mental Health Scale: Validation and measurement invariance across eight countries, genders, and age groups.	Psychological Assessment						X	Procedure described by Svetina et al. (2020).	8	6	8302
Encantado J.	Development and cross-cultural validation of the Goal Content for Weight Maintenance Scale (GCWMS)	Eating Weight Disord.			X	Separate CFA	Back and forth translation, expert review	X	likelihood ratio test	3	3	1511
Luquiens A.	Development of the Alcohol Quality of Life Scale (AQoLS): a new patient-reported outcome measure to assess health-related quality of life in alcohol use disorder	Qual. Life Res.	X	Concept elicitation focus groups; expert review	X	Cognitive interview				2	1	38
Gibbons, Robert D	Successful validation of the CAT-MH Scales in a sample of Latin American migrants in the United States and Spain.	Psychological Assessment					Matías-Carrelo et al., 2003	X	DIF	2	2	2074
Louis J.P.	Psychometric validation of the Young Parenting Inventory - Revised (YPI-R2): Replication and Extension of a commonly used parenting scale in Schema Therapy (ST) research and practice	PLoS ONE						X	MGCFA	4	1	3074
Joshanloo, Mohsen	Cross-cultural validation of fear of happiness scale across 14 national groups.	Journal of Cross-Cultural Psychology					Back and forth translation	X	MGCFA	14	6	2398
Von Steinbuchel N.	Quality of life after brain injury (QOLIBRI): Scale development and metric properties	J. Neurotrauma	X	consensus meetings of an international consortium	X	separate internal reliability	Acquadro et al., 1996).			9	6	2449
del Libano, Mario	Validity of a brief workaholism scale.	Psicothema						X	MGCFA	2	2	2714
Rye M.	The Evidence-based Practice Attitude Scale-36 (EBPAS-36): a brief and pragmatic measure of attitudes to evidence-based practice validated in US and Norwegian samples	Implement Sci			X	Separate CFA	Back and forth translation			2	2	1256
Figueroa R.	The Obesity Parenting Intervention Scale: Factorial Validity and Invariance Among Head Start Parents	Am. J. Prev. Med.					team consensus	X	MGCFA	N/A	2	578
Llosa A.E.	Development of a patient rated scale for mental health global state for use during humanitarian interventions	Int J Methods Psychiatr Res	X	Consensus group and community focus group	X	Separate PCA in each language	Back and forth translation			2	2	158
Kueh Y.C.	Cross-cultural validation of the decisional balance scale in exercise across countries	PLoS ONE						X	Byrne guideline	2	2	1136
Sproesser, Gudrun	The Positive Eating Scale: Relationship with objective health parameters and validity in Germany, the USA and India.	Psychology & Health			X	Separate CFA	Douglas and Craig 2007 collaborative and interative translation			3	3	749

Lieven, Theo	Global validation of the Coronavirus Anxiety Scale (CAS).	Curr Psychol					Back and forth translation	X	MGCFA	25	15	10232
Fujino Y.	Cross-cultural validation of the work functioning impairment scale (WFun) among Japanese, English, and Chinese versions using Rasch analysis	J Occup Health						X	Differential item functioning (DIF) and differential test functioning (DTF)	3	3	1700
Wang K.T.	The Cross-Cultural Loss Scale: development and psychometric evaluation	Psychol Assess	X	Individual interview						1	1	262
Rochefort C.	Evaluating the Validity of the Risk Perception Survey for Developing Diabetes Scale in a Safety-Net Clinic Population of English and Spanish Speakers	Diabetes Educ					Bilingual staff review	X	MGCFA	1	2	641
Pacheco, Paola Ochoa	The Working Conditions Questionnaire: Cross-cultural validation and scale refinement in six Ibero-American countries.	Employee Relations						X	MGCFA	6	1	7404
Lopez-Fernandez O.	Cross-Cultural Validation of the Compulsive Internet Use Scale in Four Forms and Eight Languages	Cyberpsychol Behav Soc Netw					Back and forth translation	X	MGCFA	15	8	4226
Vecchione, Michele	The Perceived Political Self-Efficacy Scale-Short Form (PPSE-S): A validation study in three Mediterranean countries.	Cross-Cultural Research: The Journal of Comparative Social Science					Back and forth translation	X	MGCFA	3	3	1321
Stevelink S.A.	Development and structural validation of a shortened version of the Participation Scale	Disabil Rehabil			X	Separate EFA and CFA for each dataset				6	6	5125
Hepperlen R.A.	Evaluating the cross-cultural validity of three family quality of life sub-scales	J Appl Res Intellect Disabil						X	DIF	2	N/A	1444
Cengiz H.	Death-Related Status Consumption: Scale Development and Validation	Omega (Westport)			X	Separate CFA				2	1	1302
Chen P.-Y.	Validating the cross-cultural factor structure and invariance property of the Insomnia Severity Index: Evidence based on ordinal EFA and CFA	Sleep Med.						X	MGCFA	3	2	2820
Romera, Eva M	How much do adolescents cybergossip? Scale development and validation in Spain and Colombia.	Frontiers in Psychology						X	MGCFA	2	1	3747
Zhou, Xiang	Construction and cross-cultural validation of parenting regulatory focus scale.	Dissertation Abstracts International: Section B: The Sciences and Engineering					Brislin	X	MGCFA	2	2	853
Boivin J.	The fertility quality of life (FertiQoL) tool: Development and general psychometric properties	Hum. Reprod.	X	Focus group			Back and forth translation			10	20	525
Egolf A.	Cross-cultural validation of the short version of the Food Disgust Scale in ten countries	Appetite					Back and forth translation	X	MGCFA	10	5	6128
Michaud, James	Development and validation of the Leadership Effectiveness in Africa and the Diaspora (LEAD) Scale.	International Journal of Cross Cultural Management	X	Delphi and focus group	X	Measurement refinement in each setting		X	MGCFA	19	1	1384
Ndosi M.	Cross-cultural validation of the Educational Needs Assessment Tool in RA in 7 European countries	BMC Musculoskelet. Disord.					Beaton's cross-cultural adaptation process	X	Rasch analysis and DIF	7	7	1042
Knoll, Michael	International differences in employee silence motives: Scale validation, prevalence, and relationships with culture characteristics across 33 countries.	Journal of Organizational Behavior					Brislin	X	MGCFA	33	21	8222

Vogel, David L	Cross-cultural validation of the Perceptions of Stigmatization by Others for Seeking Help (PSOSH) Scale.	Stigma and Health					Back and forth translation	X	sequential constraint imposition approach	11	N/A	3440
Lau, Chloe	Measuring Italian resilience: Cross-cultural validity and psychometric properties of the Essential Resilience Scale in Italian and Canadian contexts.	European Journal of Psychological Assessment					standardized test translation procedures	X	MGCFA	2	2	1394
Schlosser, Oliver	Humane orientation as a new cultural dimension of the GLOBE project: A validation study of the GLOBE Scale and out-group humane orientation in 25 countries.	Journal of Cross-Cultural Psychology			X	Separate CFA	Brislin			25	N/A	876
Olafsen, Anja Hagen	The Basic Psychological Need Satisfaction and Need Frustration at Work Scale: A Validation Study.	Front Psychol					Brislin	X	MGCFA	2	2	1432
Gille, Vera	Stress-symptoms and well-being in children and adolescents: Factor structure, measurement invariance, and validity of English, French, German, Russian, Spanish, and Ukrainian language versions of the SSKJ scales.	Health Psychology and Behavioral Medicine			X	Separate CFA	Back and forth translation	X	MGCFA	10	6	5227
Luengo Kanacri, Bernadette Paula	Measuring Prosocial Behaviors: Psychometric Properties and Cross-National Validation of the Prosociality Scale in Five Countries.	Front Psychol			X	Separate CFA	Back and forth translation	X	MGCFA	5	5	1630
Wambogo E.A.	Validity of the Food Insecurity Experience Scale (FIES) for use in Sub-Saharan Africa (SSA)	FASEB J.						X	Rasch analysis	36	N/A	57792
Zheng, Xiaoming	Employee well-being in organizations: Theoretical model, scale development, and cross-cultural validation.	Journal of Organizational Behavior						X	MGCFA	2	N/A	674
Tze, Virginia M. C	A cross-cultural validation of the Learning-Related Boredom Scale (LRBS) with Canadian and Chinese college students.	Journal of Psychoeducational Assessment					Back and forth translation	X	MGCFA	2	2	405
Pendergast L.L.	Assessing development across cultures: Invariance of the Bayley-III Scales Across Seven International MAL-ED sites	Sch Psychol Q					Back and forth translation, expert review	X	multiple indicator multiple causes (MIMIC)	8	8	1867
Pendergast, Laura L	Approach temperament across cultures: Validity of the Infant Temperament Scale in MAL-ED.	International Journal of School & Educational Psychology					Back and forth translation	X	multiple indicator multiple causes (MIMIC) + DIF	8	8	1933
Hong, Philip Young P	A cross-national validation of the Short Employment Hope Scale (EHS-14) in the United States and South Korea.	Social Work Research					Back and forth translation	X	MGCFA	2	2	848
Spagnoli, Paola	Factorial validity, cross-cultural equivalence, and latent means examination of the Organizational Career Growth scale in Italy and China.	The International Journal of Human Resource Management					Back and forth translation	X	MGCFA	2	2	1138
Spagnoli, Paola	A contribution toward the adaptation and validation of the entrepreneurial self-efficacy scale in Italy and Portugal.	Journal of Career Assessment					Back and forth translation	X	MGCFA	2	2	815
Young S.L.	The Individual Water Insecurity Experiences (IWISE) Scale: Reliability, equivalence and validity of an individual-level measure of water security	BMJ Glob. Health	X	Adaptation from a relevant work that have review	X	Cognitive interview	Translator review of translation	X	MGCFA; Criterion validity	31	59	43970
Gilworth G.	Adaptation and cross-cultural validation of the RA-WIS (Work Instability Scale) (Annals of the Rheumatic Diseases (2009) 68, (1686-1690))	Ann. Rheum. Dis.					Back and forth translation, expert review	X	Rasch analysis and DIF	4	4	306

Vogel D.L.	Cross-cultural validity of the self-stigma of seeking help (SSOSH) scale: Examination across six nations	J. Couns. Psychol.					Back and forth translation, expert review	X	MGCFA (sequential constraint imposition approach)	6	5	3585
Smolenski D.J.	Revision, criterion validity, and multigroup assessment of the reactions to homosexuality scale	J Pers Assess						X	Nested model comparison	N/A	2	3125
Lorenz, Timo	Validation of the revised Compound PsyCap Scale (CPC-12R) and its measurement invariance across the US and Germany.	Front Psychol						X	MGCFA	2	2	587
Boudjemadi, Valerian	Assessing the cross-cultural validity of the Succession, Identity and Consumption (SIC) Scale across four French-speaking countries.	International Review of Social Psychology						X	MGCFA and MIMIC	4	1	1827
Geyh S.	Cross-cultural validity of four quality of life scales in persons with spinal cord injury	Health Qual. Life Outcomes						X	DIF	6	3	243
Dinic, Bojana M	Cross-cultural comparison of the Benign and Malicious Envy Scale (BeMaS) across Serbian and US samples and further validation.	European Journal of Psychological Assessment						X	DIF	2	1	417
Lau, Chloe	How essential is the essential resilience scale? Differential item functioning of Chinese and English versions and criterion validity.	Personality and Individual Differences						X	DIF	2	2	753
Suhonen R.	Cross-cultural validity of the Individualised Care Scale - A Rasch model analysis	J. Clin. Nurs.						X	Rasch analysis	3	N/A	1093
Tran H.	Structural Validation and Multiple Group Assessment of the Short Internalized Homonegativity Scale in Homosexual and Bisexual Men in 38 European Countries: Results From the European MSM Internet Survey	J Sex Res						X	Nested model	33	N/A	130718
Maltby J.	The Development of a Quality of Life Scale for Informal Carers for Older Adults	Gerontol. Geriatr. Med.			X	Separate CFA				2	2	295
Akoto, Edward Osei	Cross-cultural factorial validity of the academic motivation scale.	Cross Cultural Management						X	MGCFA	2	1	529
Johnston, Claire S	Validation of an adapted French form of the Career Adapt-Abilities Scale in four Francophone countries.	Journal of Vocational Behavior						X	MGCFA	4	1	1707
Haroz E.E.	Measuring Hope Among Children Affected by Armed Conflict: Cross-Cultural Construct Validity of the Children's Hope Scale	Assessment					Van Ommeren	X	MGCFA	3	3	1057
Boivin J.	The Fertility Quality of Life (FertiQoL) tool: Development and general psychometric properties	Fertil. Steril.	X	Cross-country expert panel, focus group discussion						5	1	1414
Sorgente, Angela	The Financial Identity Scale (FIS): A multinational validation and measurement invariance study among emerging adults.	International Journal of Behavioral Development					Back and forth translation	X	MGCFA	3	3	2411
Wilson, Claire A	Resiliency across cultures: A validation of the Resiliency Scale for Young Adults.	Journal of Psychoeducational Assessment					Expert translation	X	Nested model	2	2	548
Cerin E.	Sharing good NEWS across the world: developing comparable scores across 12 countries for the Neighborhood Environment Walkability Scale (NEWS)	BMC Public Health			X	Separate CFA	Back and forth translation			12	N/A	14305
Coelho, Gabriel L. H	Optimism scale: Evidence of psychometric validity in two countries and correlations with personality.	Personality and Individual Differences						X	MGCFA (MLR)	2	N/A	746
Choi, Hyung-Min	Hotel Environmental Management Initiative (HEMI) scale development.	International Journal of Hospitality Management			X	Separate CFA	Back and forth translation	X	invariance test	2	2	595

Hjemdal, Odin	The cross-cultural validity of the Resilience Scale for Adults: a comparison between Norway and Brazil.	BMC Psychol						X	MGCFA	2	2	536
Meng, Juan (Gloria)	Technology Readiness Index (TRI): Assessing cross-cultural validity.	Journal of International Consumer Marketing					Back and forth translation	X	MGCFA	2	2	468
Carrozzino D.	Cross-cultural validity of the WHO-5 Well-Being Index and Euthymia Scale: A clinimetric analysis	J. Affective Disord.						X	Mokken and Rasch analyses	5	5	3762
Vaingankar J.A.	Development of the Chinese, Malay and Tamil translations of the Positive Mental Health Instrument: Cross-cultural adaptation, validity and internal consistency	Transcult. Psychiatry					Back and forth translation, expert review	X	DIF	1	3	660
Ford, John B	The cross-cultural scale development process: The case of brand-evoked nostalgia in Belgium and the United States.	Journal of Business Research						X	MGCFA	2	N/A	660
Zanon, Cristian	Validation of the Satisfaction with Life Scale to Brazilians: Evidences of measurement noninvariance across Brazil and US.	Social Indicators Research					Back and forth translation	X	MGCFA	2	2	1629
Sobral M.P.	COMPI fertility problem stress scales is a brief, valid and reliable tool for assessing stress in patients seeking treatment	Hum. Reprod.					Back and forth translation	X	MGCFA	7	7	3923
Bloemeke J.	Cross-cultural selection and validation of instruments to assess patient-reported outcomes in children and adolescents with achondroplasia	Qual. Life Res.	X	Literature based review, focus group discussion	X	Cognitive debrefing				2	2	133
Jasielska, Dorota	General trust scale: Validation in cross-cultural settings.	Current Psychology: A Journal for Diverse Perspectives on Diverse Psychological Issues					Back and forth translation, expert review	X	MGCFA	2	2	433
Giromini L.	Cross-Cultural Validation of the Rorschach Developmental Index	J Pers Assess						X	Separate correlation in each sample	2	2	902
Aresi, Giovanni	Development and validation of a Multidimensional Motivations to Study Abroad Scale (MMSAS) among European Credit Mobility Students.	International Journal of Intercultural Relations	X	Literature based review, pilot qualitative study, expert review	X	Separate CFA	Brislin	X	MGCFA	43	5	1333
Chen, Yu-Ping	Family role performance: Scale development and validation.	Applied Psychology: An International Review	X	In-depth interview with cross-country sample	X	covariance structure approach	Back and forth translation			22	2	534
Mezquita, Laura	Preliminary validity and reliability evidence of the Brief Antisocial Behavior Scale (B-ABS) in young adults from four countries.	PLoS ONE					Back and forth translation	X	DIF	4	3	3188
Schulze D.	Comparative organizational research starts with sound measurement: Validity and invariance of Turker's corporate social responsibility scale in five cross-cultural samples	PLoS ONE					Back and forth translation	X	MGCFA, BSEM	3	2	1220
Onwezen, Marleen C	Development and cross-cultural validation of a shortened social representations scale of new foods.	Food Quality and Preference			X	Separate CFA	Back and forth translation			3	3	1496
Streib, Heinz	The Religious Schema Scale: Construction and initial validation of a quantitative measure for religious styles.	International Journal for the Psychology of Religion					Back and forth translation	X	MGCFA	2	2	822
Boer M.	Cross-national validation of the social media disorder scale: findings from adolescents from 44 countries	Addiction					Back and forth translation, expert review	X	MGCFA	44	N/A	222532
Shoychet, Gillian	Assessing positive adaptation during a global crisis: The development and validation of the family positive adaptation during COVID-19 scale.	Front Psychol						X	MGCFA	4	1	549

Ruan, Jiening	Cross-cultural validation of Teachers' Sense of Efficacy Scale in three Asian countries: Test of measurement invariance.	Journal of Psychoeducational Assessment			X	Separate CFA	Back and forth translation	X	MGCFA	3	3	489
Partsch, Melanie V	Measuring self-control in international large-scale surveys: Development and validation of a four-item scale in English, French, German, Japanese, Polish, and Spanish.	European Journal of Psychological Assessment					TRAPD approach	X	MGCFA	6	6	5557
Villiers-Tuthill A.	Development and Validation of a Cross-Country Hospital Patient Quality of Care Assessment Tool in Europe	Patient	X	Focus group, expert panel			Back and forth translation			7	7	531
Napolitano C.M.	Assessing the implicit theory of willpower for strenuous mental activities scale: Multigroup, across-gender, and cross-cultural measurement invariance and convergent and divergent validity	Psychol. Assess.					Two independent translation	X	MGCFA	3	3	2781
Cleveland, Mark	Cross-linguistic validation of a unidimensional scale for cosmopolitanism.	Journal of Business Research						X	MGCFA	4	7	1538
Komissarouk, Svetlana	Dispositional differences in seeking autonomy- or dependency-oriented help: Conceptual development and scale validation.	Personality and Individual Differences			X	Separate CFA	Back and forth translation			3	3	486
Fischmann, Gabriel	Validation of a short and generic Qualitative Job Insecurity Scale (QUAL-JIS).	European Journal of Psychological Assessment						X	MGCFA	2	2	2380
Eisma M.C.	Development and psychometric evaluation of the Utrecht grief rumination scale	J. Psychopathol. Behav. Assess.			X	Separate CFA	Back and forth translation	X	MGCFA	2	2	520
Chow T.S.	Family Resilience Scale Short Form (FRS16): Validation in the US and Chinese Samples	Front. Psychiatry			X	Separate CFA		X	MGCFA	2	2	2371
Afulani P.A.	Toward the development of a short multi-country person-centered maternity care scale	Int. J. Gynecol. Obstet.			X	Separate CFA				3	6	3663
Nackers F.	A screening tool for psychological difficulties in children aged 6 to 36 months: Cross-cultural validation in Kenya, Cambodia and Uganda	BMC Pediatr.	X	Cross-cultural expert panel	X	Separate EFA for each dataset	Back and forth translation, expert review			3	3	824
Caycho-Rodriguez, Tomas	Cross-cultural validation of the new version of the Coronavirus Anxiety Scale in twelve Latin American countries.	Curr Psychol			X	Separate CFA		X	MGCFA	12	1	5196
Pepe, Silvia J	Work Self-Efficacy Scale and Search for Work Self-Efficacy Scale: A validation study in Spanish and Italian cultural contexts.	Revista de Psicologia del Trabajo y de las Organizaciones						X	MGCFA	2	2	658
Reyes-Bossio, Mario	Cross-cultural validity of the five items Mindful Attention Awareness Scale (MAAS-5) in Peru and Mexico during the COVID-19 pandemic.	Psicol. reflex. crit.						X	MGCFA	2	2	1144
Whiting-Collins L.	Measuring contraceptive self-efficacy in sub-Saharan Africa: development and validation of the CSESSA scale in Kenya and Nigeria	Contraception X	X	Literature based review, focus group discussion	X	Separate PCA, reliability and validty test in each sample				2	6	728
Schutte, Lusilda	Problematic factorial validity of three language versions of the Basic Psychological Needs Scale (BPNS): Why and what are the implications?	Journal of Happiness Studies: An Interdisciplinary Forum on Subjective Well-Being			X	Separate CFA		X	MGCFA	1	3	1056
Louis J.P.	The Development of the Love and Respect Marriage Scale	J Sex Marital Ther						X	MGCFA	5	N/A	1856
Khan, Anzalee	A rasch model to test the cross-cultural validity in the positive and negative syndrome scale (PANSS) across six geo-cultural groups.	BMC Psychol						X	Rasch analysis and DIF	27	N/A	1169

Heikamp, Tobias	Cross-cultural validation of the positivity-scale in five European countries.	Personality and Individual Differences					Back and forth translation	X	MGCFA	5	5	3544
Goroshit, Marina	Life-domain regret regarding procrastination (LDR-P): Scale validation in the United States and Israel.	Current Psychology: A Journal for Diverse Perspectives on Diverse Psychological Issues			X	Separate CFA	Back and forth translation	X	MGCFA	2	2	3197
Li J.-B.	The Parent-Version of the Spence Children's Anxiety Scale (SCAS-P) in Chinese and Italian Community Samples: Validation and Cross-Cultural Comparison	Child Psychiatry Hum Dev						X	MGCFA	2	2	908

Appendix S4. Commonly used indices for CFA and MGCFA

For CFA

**title**	**CFI**	**RMSEA**	**SRMR**	**TFI**
The Good Mothering Expectations Scale: An international instrument development study.				
Cross-cultural search for Big Five: Development of a scale to compare personality traits of pre-service elementary school teachers in Turkey and Spain.	>0.90	<0.08	<0.08	
Stress measurement among charge nurses: developing a cross-cultural tool	>0.90	<0.08		
The development of the Compulsive Sexual Behavior Disorder Scale (CSBD-19): An ICD-11 based screening measure across three languages	>=0.90	<=0.08		
Development and cross-cultural validation of the Goal Content for Weight Maintenance Scale (GCWMS)	>0.90	<0.05	<0.05	
The Evidence-based Practice Attitude Scale-36 (EBPAS-36): a brief and pragmatic measure of attitudes to evidence-based practice validated in US and Norwegian samples	>0.95	<0.06	<0.08	
The Positive Eating Scale: Relationship with objective health parameters and validity in Germany, the USA and India.	>=0.90	<=0.08	<=0.10	
Development and structural validation of a shortened version of the Participation Scale	>0.95	<0.08		>0.95
Death-Related Status Consumption: Scale Development and Validation				
Humane orientation as a new cultural dimension of the GLOBE project: A validation study of the GLOBE Scale and out-group humane orientation in 25 countries.				>0.90
Stress-symptoms and well-being in children and adolescents: Factor structure, measurement invariance, and validity of English, French, German, Russian, Spanish, and Ukrainian language versions of the SSKJ scales.	>=0.95	<=0.06	<=0.08	
Measuring Prosocial Behaviors: Psychometric Properties and Cross-National Validation of the Prosociality Scale in Five Countries.	>0.90	<0.08		>0.90
The Development of a Quality of Life Scale for Informal Carers for Older Adults	>=0.90	<0.08	<0.08	
Sharing good NEWS across the world: developing comparable scores across 12 countries for the Neighborhood Environment Walkability Scale (NEWS)	>=0.90	<=0.06	<=0.08	
Hotel Environmental Management Initiative (HEMI) scale development.				
Development and validation of a Multidimensional Motivations to Study Abroad Scale (MMSAS) among European Credit Mobility Students.	>0.90	<0.08	<0.08	
Development and cross-cultural validation of a shortened social representations scale of new foods.	>=0.95	<=0.06	<=0.08	
Cross-cultural validation of Teachers' Sense of Efficacy Scale in three Asian countries: Test of measurement invariance.	>0.95	<0.05	<0.08	
Dispositional differences in seeking autonomy- or dependency-oriented help: Conceptual development and scale validation.	>=0.95	<=0.07		
Development and psychometric evaluation of the Utrecht grief rumination scale	>0.90	<=0.08		>0.90
Family Resilience Scale Short Form (FRS16): Validation in the US and Chinese Samples				
Toward the development of a short multi-country person-centered maternity care scale				
Cross-cultural validation of the new version of the Coronavirus Anxiety Scale in twelve Latin American countries.	>0.95	<0.08	<0.08	>0.90

Problematic factorial validity of three language versions of the Basic Psychological Needs Scale (BPNS): Why and what are the implications?	>=0.95	<0.08		
Life-domain regret regarding procrastination (LDR-P): Scale validation in the United States and Israel.				

For MGCFA

title	Min sample in sub-group	ΔCFI	ΔRMSEA	ΔSRMR for metric	ΔSRMR for scalar	ΔTLI	ΔNCL	Other
Why do people use new psychoactive substances? Development of a new measurement tool in six European countries	272	<0.01	<0.015					
Development and validation of a health and nutrition module for the project-level Women's Empowerment in Agriculture Index (pro-WEAI+HN)								
Going your own way: A cross-cultural validation of the Motivational Demands at Work Scale (mind@work).	308							
A cross-cultural validation of the Resiliency Scale for Young Adults in Canada and China	617	<= 0.005	<=0.01					
Psychometric validity and measurement invariance of the caring for Bliss Scale in the Philippines and the United States	546	<0.01	<0.015					
Cross cultural validation of the Yale Food Addiction Scale 2.0 and evaluation of food addiction in Hispanic bariatric surgery-seeking patients.	215							
Testing the Situationism Scale in Europe: Scale validation, self-regulation and regional differences	62							
Examining the validity, reliability, and measurement invariance of the Social Support for Exercise Scale among Spanish- and English-language Hispanics.	369	<=0.01	<0.01	<0.025	<0.005			
Perceived collective efficacy to stop aggression at school: A validation of an Italian and a Swedish version of a scale for adolescents.	126							
Measuring parental anchoring: The development and validation of the Parental Anchoring Scale.	172							
Cross-cultural measurement invariance in the Satisfaction with Food-Related Life Scale in older adults from two developing countries.	840							
Cross-cultural validation of the Worries about COVID-19 and its consequences Scale (W-COV) in adolescents and young people	334	<0.01	<0.015	<0.03				
Measurement invariance of English and French Health Education Impact Questionnaire (heiQ) empowerment scales validated for cancer	520	<0.01	<=0.015					
Consumer arrogance: Scale development and validation.	149							
The Multidimensional Work Motivation Scale: Validation evidence in seven languages and nine countries.	530	<=0.01						

Refinement of the Analysis-Holism Scale: A cross-cultural adaptation and validation of two shortened measures of analytic versus holistic thinking in Spain and the United States.	295	<0.01	<0.015					
Validation of the Procrastination at Work Scale: A seven-language study.	126	<=0.01						
Validation of the GEOPTE scale of social cognition for psychosis in patients diagnosed with schizophrenia in Latin America.	83	<0.01	<0.01					
Professional development short scale: Measurement invariance, stability, and validity in Brazil and Angola.	546	<0.01						
Cross-cultural validation of the Basic Psychological Needs in Physical Education Scale between Portugal and Brazil samples.	450	<=0.01						
Cross-Cultural Validity of the Ruminative Responses Scale in Argentina and the United States	308	<0.01	<0.015	<0.03	<=0.01			
Cross-cultural validity of Morningness-Eveningness Stability Scale improved (MESSi) in Iran, Spain and Germany	193	<0.01						
The development of the Compulsive Sexual Behavior Disorder Scale (CSBD-19): An ICD-11 based screening measure across three languages	380	<=0.01	<=0.015			<=0.01		
Psychometric validation of the Young Parenting Inventory - Revised (YPI-R2): Replication and Extension of a commonly used parenting scale in Schema Therapy (ST) research and practice	204							
Cross-cultural validation of fear of happiness scale across 14 national groups.	85							
Validity of a brief workaholism scale.	550							
The Obesity Parenting Intervention Scale: Factorial Validity and Invariance Among Head Start Parents	130	<0.015	<0.015	<0.03	<0.015			
Global validation of the Coronavirus Anxiety Scale (CAS).	394							
Evaluating the Validity of the Risk Perception Survey for Developing Diabetes Scale in a Safety-Net Clinic Population of English and Spanish Speakers	269							$\chi2$
The Working Conditions Questionnaire: Cross-cultural validation and scale refinement in six Ibero-American countries.	371	<0.005	<0.01	<0.025	<0.005			
Cross-Cultural Validation of the Compulsive Internet Use Scale in Four Forms and Eight Languages	258	<=0.01	<=0.015	<=0.03	<=0.01			
The Perceived Political Self-Efficacy Scale-Short Form (PPSE-S): A validation study in three Mediterranean countries.	354	<=0.01	<=0.015					
Validating the cross-cultural factor structure and invariance property of the Insomnia Severity Index: Evidence based on ordinal EFA and CFA	354	<=0.01						
How much do adolescents cybergossip? Scale development and validation in Spain and Colombia.	1816	<=0.01	<=0.02	<=0.03				
Construction and cross-cultural validation of parenting regulatory focus scale.	356							$\chi2$

Cross-cultural validation of the short version of the Food Disgust Scale in ten countries	572	<=0.01	<=0.015					
Development and validation of the Leadership Effectiveness in Africa and the Diaspora (LEAD) Scale.	441	<=0.01						χ2
International differences in employee silence motives: Scale validation, prevalence, and relationships with culture characteristics across 33 countries.	157	<=0.01	<=0.015					χ2
Measuring Italian resilience: Cross-cultural validity and psychometric properties of the Essential Resilience Scale in Italian and Canadian contexts.	500	<=0.01	<=0.015					
The Basic Psychological Need Satisfaction and Need Frustration at Work Scale: A Validation Study.	281	<0.01	<0.015	<0.03	<0.015			
Stress-symptoms and well-being in children and adolescents: Factor structure, measurement invariance, and validity of English, French, German, Russian, Spanish, and Ukrainian language versions of the SSKJ scales.	207	<=0.01					<=0.02	
Measuring Prosocial Behaviors: Psychometric Properties and Cross-National Validation of the Prosociality Scale in Five Countries.	116	<=0.01						
Employee well-being in organizations: Theoretical model, scale development, and cross-cultural validation.	250							
A cross-cultural validation of the Learning-Related Boredom Scale (LRBS) with Canadian and Chinese college students.	151	<=0.01						χ2
A cross-national validation of the Short Employment Hope Scale (EHS-14) in the United States and South Korea.	390							χ2
Factorial validity, cross-cultural equivalence, and latent means examination of the Organizational Career Growth scale in Italy and China.	256	<=0.01	<=0.015					
A contribution toward the adaptation and validation of the entrepreneurial self-efficacy scale in Italy and Portugal.	123	<=0.01	<=0.015					
The Individual Water Insecurity Experiences (IWISE) Scale: Reliability, equivalence and validity of an individual-level measure of water security	878	<=0.01	<=0.02					Aligment methods
Cross-cultural validity of the self-stigma of seeking help (SSOSH) scale: Examination across six nations	299	<=0.02						
Revision, criterion validity, and multigroup assessment of the reactions to homosexuality scale	733	<=0.01						
Validation of the revised Compound PsyCap Scale (CPC-12R) and its measurement invariance across the US and Germany.	202	<0.01	<0.015	<0.03	<0.015			
Assessing the cross-cultural validity of the Succession, Identity and Consumption (SIC) Scale across four French-speaking countries.	385	<=0.01						
Structural Validation and Multiple Group Assessment of the Short Internalized Homonegativity Scale in Homosexual and Bisexual Men in 38 European Countries: Results From the European MSM Internet Survey	10490	<=0.01	<=0.015					
Cross-cultural factorial validity of the academic motivation scale.	262	<=0.01						

Validation of an adapted French form of the Career Adapt-Abilities Scale in four Francophone countries.	181	<0.01	<0.05					
Measuring Hope Among Children Affected by Armed Conflict: Cross-Cultural Construct Validity of the Children's Hope Scale	325							χ2
The Financial Identity Scale (FIS): A multinational validation and measurement invariance study among emerging adults.	481	<0.01	<0.015					
Resiliency across cultures: A validation of the Resiliency Scale for Young Adults.	259							χ2
Optimism scale: Evidence of psychometric validity in two countries and correlations with personality.	325	<=0.01		<=0.03				
The cross-cultural validity of the Resilience Scale for Adults: a comparison between Norway and Brazil.	222							
Technology Readiness Index (TRI): Assessing cross-cultural validity.	231							χ2
The cross-cultural scale development process: The case of brand-evoked nostalgia in Belgium and the United States.	188							
Validation of the Satisfaction with Life Scale to Brazilians: Evidences of measurement noninvariance across Brazil and US.	241							χ2
COMPI fertility problem stress scales is a brief, valid and reliable tool for assessing stress in patients seeking treatment	96	<0.01	<0.015					
General trust scale: Validation in cross-cultural settings.	203							Alignment methods
Development and validation of a Multidimensional Motivations to Study Abroad Scale (MMSAS) among European Credit Mobility Students.	83	<=0.01	<=0.015					
Comparative organizational research starts with sound measurement: Validity and invariance of Turker's corporate social responsibility scale in five cross-cultural samples	146	<0.01	<0.015	<0.03	<0.005			
The Religious Schema Scale: Construction and initial validation of a quantitative measure for religious styles.	255							χ2
Cross-national validation of the social media disorder scale: findings from adolescents from 44 countries		<=0.01	<=0.015					
Assessing positive adaptation during a global crisis: The development and validation of the family positive adaptation during COVID-19 scale.	136							χ2
Cross-cultural validation of Teachers' Sense of Efficacy Scale in three Asian countries: Test of measurement invariance.	879	<0.01	<0.015	<0.03	<0.01			
Measuring self-control in international large-scale surveys: Development and validation of a four-item scale in English, French, German, Japanese, Polish, and Spanish.	1144	<0.01						χ2

Assessing the implicit theory of willpower for strenuous mental activities scale: Multigroup, across-gender, and cross-cultural measurement invariance and convergent and divergent validity	302							χ2
Cross-linguistic validation of a unidimensional scale for cosmopolitanism.	388	<=0.01	<=0.015					
Validation of a short and generic Qualitative Job Insecurity Scale (QUAL-JIS).	204							χ2
Development and psychometric evaluation of the Utrecht grief rumination scale	1135	<=0.01	<=0.015					
Family Resilience Scale Short Form (FRS16): Validation in the US and Chinese Samples	163							χ2
Cross-cultural validation of the new version of the Coronavirus Anxiety Scale in twelve Latin American countries.	253	<=0.01	<=0.015					χ2
Work Self-Efficacy Scale and Search for Work Self-Efficacy Scale: A validation study in Spanish and Italian cultural contexts.	203							χ2
Cross-cultural validity of the five items Mindful Attention Awareness Scale (MAAS-5) in Peru and Mexico during the COVID-19 pandemic.	322	<0.01						
Problematic factorial validity of three language versions of the Basic Psychological Needs Scale (BPNS): Why and what are the implications?	256	<=0.01	<=0.015					
The Development of the Love and Respect Marriage Scale	306							χ2
Cross-cultural validation of the positivity-scale in five European countries.	320	<=0.01						χ2
Life-domain regret regarding procrastination (LDR-P): Scale validation in the United States and Israel.	897	<=0.01						
The Parent-Version of the Spence Children's Anxiety Scale (SCAS-P) in Chinese and Italian Community Samples: Validation and Cross-Cultural Comparison	452	<0.01						
